# A study on the influence of family social capital on participation in adolescent extracurricular sports and public health

**DOI:** 10.3389/fpubh.2025.1515522

**Published:** 2025-06-25

**Authors:** Wenjie Sui, Miaohong Huang

**Affiliations:** ^1^College of Physical Education, Huaqiao University, Quanzhou, China; ^2^Xiamen Institute of Technology, Xiamen, China

**Keywords:** public health, obesity, anxiety, depression, educational resources, CFPS data, family social capital, youth extracurricular sports

## Abstract

**Introduction:**

With increasing academic pressure, extracurricular tutoring and interest classes are growing rapidly. This trend is especially evident in youth sports interest classes. Families allocate limited resources to these activities, directly influencing their social capital. This study examines how family social capital affects adolescent participation in extracurricular sports and its broader implications for public health.

**Methods:**

This study uses longitudinal data from the China Family Tracking Survey (CFPS, 2010–2018, *N* = 12,750) to analyze the relationship between family social capital, extracurricular sports participation, and adolescent health outcomes. A Backpropagation (BP) neural network model was applied to assess the predictive power of family social capital on sports engagement. Public health indicators, such as obesity, anxiety, and depression rates, were also evaluated. Regional disparities were examined to highlight differences between urban and rural areas.

**Results:**

Adolescents from high-social-capital families had significantly higher sports participation (85%) than those from low-social-capital families (25%). They also had lower obesity rates (10% vs. 30%) and reduced anxiety and depression by 25% and 20%, respectively. Parental engagement increased the likelihood of sports participation by 15%, and educational resources positively impacted adolescent wellbeing. Regional disparities were evident, with urban adolescents participating at 70%, compared to 40% in rural areas.

**Discussion:**

The findings show that higher family social capital improves adolescents' physical and mental health. It helps reduce public health risks such as obesity, anxiety, and depression. Additionally, it increases the participation of young people in various physical activities. This study emphasizes the need to improve family social capital and address regional inequalities. These efforts can promote public health and provide equitable access to extracurricular opportunities.

## 1 Introduction

Family social capital, a crucial component of social networks, consists of the resources and support provided by family members and their connections. It plays a significant role in shaping adolescents' choices, particularly in extracurricular sports participation, which has direct implications for public health (1–3). Physical activity during adolescence is vital for preventing various health issues, including obesity, anxiety, and depression, and promoting overall wellbeing (4–6). However, disparities in family resources and social capital can lead to unequal access to extracurricular sports, affecting long-term health outcomes (7, 8).

Household income influences adolescent development through two key models: the financial capital model, which focuses on tangible resources, and the family process model, which emphasizes the household environment (9, 10). Adolescents from low-income families often face economic stress, reduced parental support, and limited access to sports activities, all of which contribute to poor physical and mental health outcomes (11–14).

As China undergoes rapid economic transformation, public health has become a critical development indicator (15, 16). While education remains a primary area of family investment, the role of extracurricular physical activities in shaping adolescent health is underexplored (17, 18). Using China Family Panel Studies (CFPS) data, researchers investigated how family social capital influences adolescent participation in extracurricular sports and its broader implications for public health (19–22).

This study makes significant contributions to public health research by demonstrating that higher family social capital leads to increased adolescent participation in extracurricular sports. This, in turn, improves physical and mental health outcomes. Our findings highlight the need for policymakers and educators to strengthen family social capital to enhance adolescent wellbeing.

The key contributions of this paper are as follows:

- This study highlights the significant role of family social capital in promoting adolescent participation in extracurricular sports and demonstrates its positive impact on both physical and mental health outcomes.
- This research emphasizes the impact of geographical location and economic conditions on family social capital, revealing regional inequalities in access to extracurricular sports opportunities and public health resources.
- The study provides actionable recommendations for policymakers and educators, focusing on enhancing family social capital as a strategy to promote healthier lifestyles and address public health challenges, such as reducing lifestyle-related health issues among adolescents.

## 2 Related work

Research on family social capital (23–27) has extensively explored its role in various domains, including education, economic mobility, and social networks. Danes et al. (23) and Hoffman et al. (25) highlighted how family capital (e.g., human, social, and financial capital) affects decision-making processes. Sorenson and Bierman (27) further emphasized that social capital within families influenced children's access to opportunities, including extracurricular activities, which had a direct impact on their wellbeing. Gofen (24) and Li (26) also underscored the role of family capital in breaking intergenerational cycles of disadvantage, particularly in education and skill development. However, the link between family social capital and adolescent extracurricular sports participation, especially its public health implications, remained underexplored. Several studies examined the impact of household income on access to extracurricular activities and overall wellbeing. Auten and Carroll (1), Jenkins (2), and Smeeding and Weinberg (3) focused on how income disparities affected household resource allocation, which directly influenced children's educational and extracurricular opportunities. Similarly, Khan et al. (8) and Stephen et al. (7) highlighted economic inequalities in China, showing that lower-income households often struggled to invest in extracurricular activities, including sports. Since physical activity was essential for reducing obesity, anxiety, and other health risks, such disparities contributed to long-term public health inequalities.

Beyond direct income effects, the relationship between economic growth, wellbeing, and social capital was explored by several scholars. Yolal et al. (10) and Lin et al. (9) found that economic transformation and urbanization influenced residents' wellbeing, often leading to shifting priorities in household expenditures. Fakfare and Wattanacharoensil (12) further highlighted that community development affected residents' satisfaction and lifestyle choices, including their engagement in extracurricular physical activities. However, these studies did not specifically address how family social capital mediated the impact of economic constraints on sports participation and adolescent health. Extracurricular physical activities played a crucial role in shaping long-term health habits and preventing lifestyle-related diseases. Pérez-Ordás et al. (17) argued that extracurricular sports contributed to sustainable development goals by promoting holistic education and wellbeing. Similarly, Bocarro et al. (19) and Cohen et al. (20) emphasized that school-based extracurricular sports provided structured opportunities for students to engage in physical activity, reducing health risks and encouraging lifelong healthy behaviors. However, participation rates varied significantly based on family social capital and economic conditions, which this study aims to examine.

Health behavior research also linked social capital to lifestyle choices. Dickinson and MacKay (21) and Suyatmin and Sukardi (22) found that individuals with stronger social networks were more likely to adopt healthier habits, suggesting that families with high social capital may have encouraged better physical activity habits among adolescents. However, these studies did not explore how family-level social capital influenced youth engagement in extracurricular sports, a key aspect of this research. Existing research highlighted how different family structures impacted household decision-making. Ivanova et al. (28) examined household consumption patterns, revealing that families allocated resources differently based on socioeconomic conditions and social capital availability. Blundell and MaCurdy (29) further explored labor supply dynamics, showing that family financial decisions directly affected educational investments, including extracurricular activities. Fredricks (30) and Weininger et al. (31) discussed how socioeconomic class influenced children's participation in extracurricular activities. They observed that money alone did not determine access. Social and cultural capital also played significant roles. Goshin et al. (32) reinforced this point by showing that parental involvement in education and extracurricular activities varied depending on their social networks and economic background. Cultural capital also played a role in extracurricular activity engagement and health outcomes. Studies by Francis and Wilcox (33), Pajares and Valiante (34), and Borges et al. (35) investigated gender orientation and its impact on educational and extracurricular participation, finding that parental expectations and cultural norms influenced students' activity choices. However, these studies did not directly address the role of family social capital in shaping participation in extracurricular sports and its effects on adolescent public health, which this study aims to explore.

## 3 Method

### 3.1 Research motivation

While previous studies have extensively examined family economic capital and cultural capital in shaping academic achievements, less attention has been given to family social capital's role in extracurricular sports and public health. The existing literature primarily focuses on factors such as household income, parents' education levels, and physical environment in determining educational and wellbeing outcomes. However, fewer studies have addressed how family social networks and parental involvement impact youth participation in physical activities outside the school curriculum.

This study aims to fill this research gap by investigating the influence of family social capital on extracurricular sports participation and its effects on adolescent public health outcomes. Using CFPS data, we examine how educational resources, parental involvement, and social capital affect adolescents' engagement in physical activities. Additionally, we explore regional disparities in social capital distribution, which impact access to extracurricular sports and create health inequalities across different socioeconomic backgrounds. Our research differs from the existing literature in several key ways. First, while previous studies have considered the role of family capital in academic success, few have specifically examined how family social capital influences extracurricular sports participation. This factor is crucial in promoting physical and mental health among adolescents. Second, by focusing on the interplay between family social capital and geographical location, we highlight the often-overlooked regional disparities that affect access to extracurricular sports opportunities and public health outcomes. Third, our study directly connects family social capital to public health issues such as obesity, anxiety, and depression. It provides empirical evidence of how enhanced family involvement can lead to better health outcomes for youth.

### 3.2 Methodology

The **Backpropagation (BP) neural network**, introduced by Buscema (38), is a supervised learning algorithm designed to optimize multi-layer neural networks (36). It iteratively adjusts network weights through error backpropagation, improving prediction accuracy (37). This study employs the BP model to assess the impact of family social capital on adolescent extracurricular sports participation and public health outcomes due to its ability to handle non-linear relationships and complex feature interactions. The key benefits of BP model can be summarized as follows [for more details (37–39)]. Unlike traditional statistical methods, BP can capture non-linear dependencies among parental engagement, household income, and regional disparities affecting sports participation. The model automatically learns hierarchical relationships, such as how family education and income jointly influence adolescent health. BP efficiently processes large datasets (e.g., 12,750 observations), thus it is ideal for analyzing social capital's role in adolescent wellbeing.

#### 3.2.1 BP network structure

**Figure 1:**
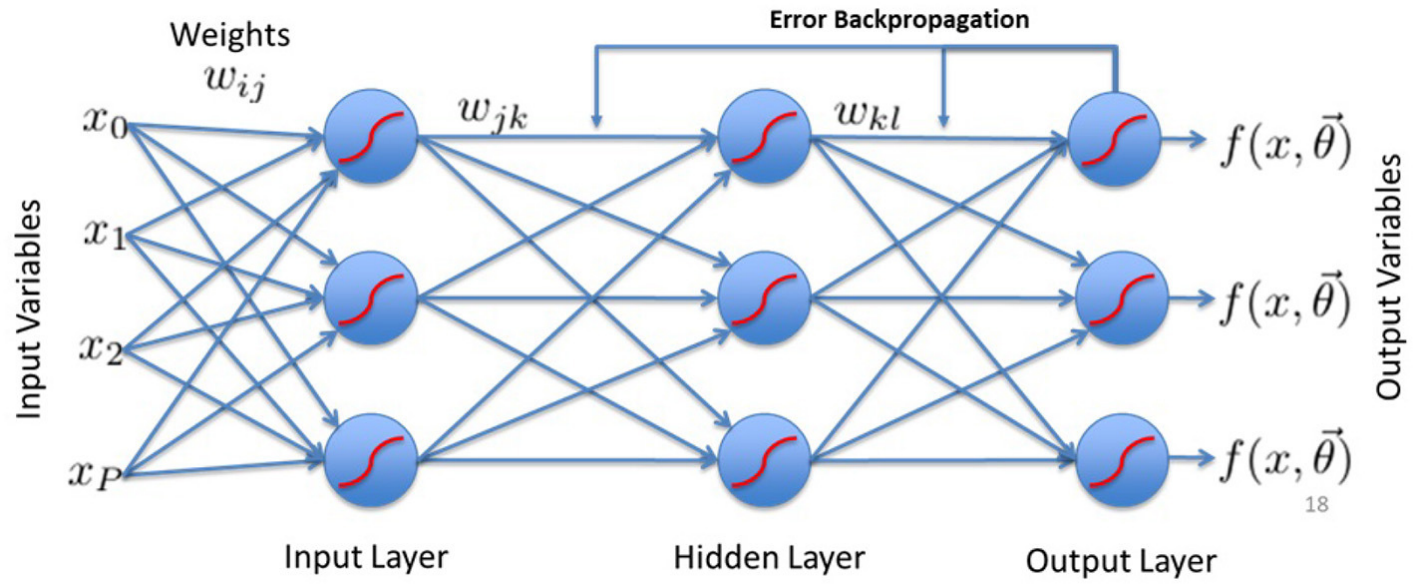
Diagrammatic flow of the BP neural network.

BP neural network used in this study consists of three primary layers, such as input layer, hidden layer, and output layer (see Figure 1). The input layer represents independent variables, including parental engagement, educational resources, and regional disparities, which influence adolescent participation in extracurricular sports. These factors serve as critical determinants in assessing the role of family social capital in shaping public health outcomes.

The hidden layer captures complex interactions between these independent variables by applying activation functions. This allows the network to model non-linear relationships, such as how a combination of family involvement and educational resources affects adolescent sports participation and mental health. Unlike traditional regression methods, which assume linear dependencies, the BP model enables a hierarchical learning process, improving predictive accuracy (37–39).

The output layer generates final predictions, including the probability of sports participation and associated health outcomes such as obesity, anxiety, and depression. By systematically adjusting connection weights through error backpropagation, the BP model refines its predictions over multiple iterations, ensuring a more reliable assessment of family social capital's impact on adolescent wellbeing. Each neuron in layer *l* connects to neurons in layer *l*−1 through weights *w*_*ij*_, dynamically adjusting based on the error feedback received during training (37–39).

#### 3.2.2 Forward sropagation

During forward propagation, the weighted sum of inputs at each neuron is computed as:


(1) $$\begin{array}{l} z_{j}^{l}=\overset{n}{\underset{i=1}{\sum }}w_{ij}^{l}x_{i}^{l-1}+b_{j}^{l} \end{array}$$

Here, $z_{j}^{l}$ represents the weighted sum of inputs at neuron *j* in layer *l*. $w_{ij}^{l}$ shows the weight between neuron *i* in layer *l*−1 and neuron *j* in layer *l*. $b_{j}^{l}$ represents bias term for neuron *j* in layer *l*.

The neuron applies an activation function to introduce non-linearity:


(2) $$\begin{array}{l} y_{j}^{l}=\frac{1}{1+e^{-z_{j}^{l}}} \end{array}$$

Here, $y_{j}^{l}$ represents the output of neuron *j* in layer *l*.

The output propagates through the layers until the final network output is computed.

#### 3.2.3 Error backpropagation

If the network output deviates from the expected target, an error signal is computed using the Mean Squared Error (MSE) loss function:


(3) $$\begin{array}{l} E=\frac{1}{2}\overset{M}{\underset{i=1}{\sum }}{\left(y_{i}-{ŷ}_{i}\right)}^{2} \end{array}$$

Here, *E* represents the total error across all training samples. *y*_*i*_ is the actual target value for sample *i*. ŷ_*i*_ is the predicted value from the network.

To minimize this error, gradient descent is used to adjust network weights:


(4) $$\begin{array}{l} \text{Δ}w_{ij}^{l}=-\eta \frac{\partial E}{\partial w_{ij}^{l}} \end{array}$$

Here, $\text{Δ}w_{ij}^{l}$ represents the weight update for neuron *j* in layer *l*. η denotes the learning rate, controlling the step size of updates.

The error term at neuron *j* in layer *l* is calculated using the chain rule:


(5) $$\begin{array}{l} \delta_{j}^{l}=\left(y_{j}^{l}-{ŷ}_{j}^{l}\right)y_{j}^{l}\left(1-y_{j}^{l}\right) \end{array}$$

(6) $$\begin{array}{l} \text{Δ}w_{ij}^{l}=-\eta \delta_{j}^{l}x_{i}^{l-1} \end{array}$$

Here, $\delta_{j}^{l}$ represents the backpropagated error at neuron *j* in layer *l*. $x_{i}^{l-1}$ denotes the output of neuron *i* in the preceding layer.

The process repeats iteratively until the error *E* is minimized below a predefined threshold ϵ.

#### 3.2.4 Modification in BP algorithm

**Figure 2:**
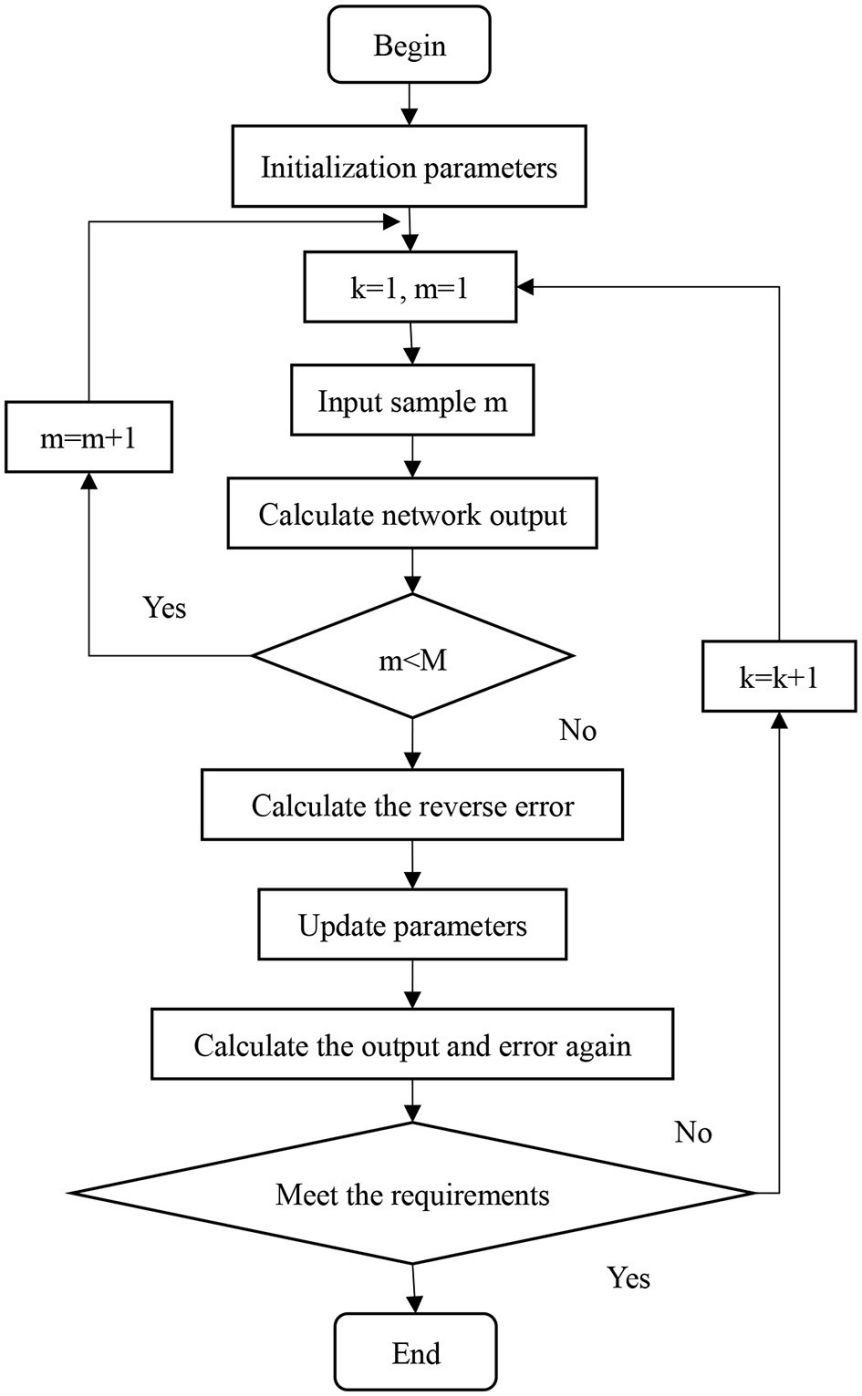
Flowchart of the enhanced BP algorithm incorporating modifications to the evaluation function and optimization strategies.

Although the BP algorithm has been widely applied in engineering, it faces certain limitations when analyzing complex or random problems. Specifically, the choice of the evaluation function significantly affects the network's performance, increasing the risk of overfitting and compromising the reliability of the results. To address these issues, several enhancements have been incorporated. It includes modifications to the evaluation function and strategies to optimize performance. The modified version of BP algorithm is shown in Figure 2.

The evaluation function includes a regularization term to balance the mean square error (MSE) and weight energy (WE) as follows:


(7) $$\begin{array}{l} reg=\alpha \cdot me+\left(1-\alpha \right)\cdot we \end{array}$$

Here, α is a balancing factor where 0 ≤ α ≤ 1. *me* refers to the mean square error between network levels, defined as:


(8) $$\begin{array}{l} me=\frac{1}{M}\overset{M}{\underset{n=1}{\sum }}\frac{{\left(z^{m}-y^{m}\right)}^{2}}{2} \end{array}$$

The weight energy (*we*) refers to the network weight regularization term and can be optimized using one of the following strategies:


(9) $$\begin{array}{l} mw_{1}=\frac{1}{C_{w}}\overset{C_{w}}{\underset{i=1}{\sum }}w_{i}^{2} \end{array}$$

(10) $$\begin{array}{l} mw_{2}=\frac{1}{C_{w}}\overset{C_{w}}{\underset{i=1}{\sum }}|w_{i}| \end{array}$$

(11) $$\begin{array}{l} mw_{3}=\frac{1}{C_{w}}\overset{C_{w}}{\underset{i=1}{\sum }}\log\left(1+\beta^{2}w_{i}^{2}\right) \end{array}$$

Here, *C*_*w*_ represents the number of adjustable neurons in the BP network. *w*_*i*_ represents the weight of the neurons in the current network layer. β is a random value that is <1.

#### 3.2.5 Optimization strategies

While BP is widely used, it encounters challenges such as overfitting and slow convergence. To enhance model performance and ensure reliable predictions, the following optimization strategies were also implemented.

Regularization ensures that the BP model does not overfit to the training data, allowing it to generalize better to unseen samples. Therefore, a regularization term is added to the loss function, which penalizes excessively large weights. This helps in reducing model complexity and improving generalization:


(12) $$\begin{array}{l} E_{\text{reg}}=E+\text{λ}\sum \overset{2}{\underset{ij}{w}} \end{array}$$

Here, *E*_reg_ represents the regularized error function. λ controls the regularization strength, where a higher value forces smaller weight values, reducing variance but potentially increasing bias.

Instead of using a fixed learning rate, an adaptive learning rate is employed to improve convergence efficiency and prevent the model from stagnating in local minima. By starting with a higher learning rate and decreasing it as training advances, the model converges faster in the initial stages and fine-tunes weights more carefully in later iterations. The learning rate is adjusted dynamically as training progresses:


(13) $$\begin{array}{l} \eta_{t}=\frac{\eta_{0}}{1+\gamma t} \end{array}$$

Here, η_*t*_ represents the learning rate at iteration *t*. γ denotes the decay factor that gradually reduces η_*t*_ over time to prevent oscillations and improve stability.

Early stopping is particularly useful when training deep networks, as excessive epochs can cause the model to memorize training data instead of learning meaningful patterns. Therefore, to avoid overfitting, training is monitored continuously, and it is stopped early if the validation loss does not improve for 10 consecutive epochs. This prevents unnecessary iterations that could lead to overfitting and ensures that the model retains optimal generalization capability.

Cross-validation minimizes bias introduced by a specific data split and provides a more reliable estimate of the model's predictive performance. It also helps in tuning hyperparameters by evaluating performance across different data partitions. In this work, a 5-fold cross-validation technique is applied to ensure robustness and generalizability of the model. The dataset is divided into five equal parts, where each subset serves as a validation set once, while the remaining four subsets are used for training. This process is repeated five times, and the final model performance is averaged across all runs.

## 4 Experimental setup

### 4.1 Data sources and variable selection

This study utilizes longitudinal data from the China Family Tracking Survey (CFPS), covering the years 2010 to 2018 across five time periods, with a total cycle span of eight years. The CFPS dataset, initiated by Peking University's Chinese Social Science Survey Center, offers comprehensive household-level information, including socioeconomic characteristics, family structure, and adolescent behavioral data.

To ensure the relevance and robustness of the dataset, the following processing principles are applied: The focus is on adolescents (**ages 10–18**) participating in extracurricular physical education. Households where the head is younger than 18 or older than 90 were excluded to maintain consistency and avoid demographic outliers. Observations with extreme values in continuous variables, such as household income (e.g., above the 99th percentile) and BMI (>40), were removed using the interquartile range (IQR) method. For BMI and mental health scores, mean imputation was used for missing continuous variables, while mode imputation was applied for binary or categorical variables. *Note:* Before data cleaning, household income ranged from 500 to 1,200,000 RMB. After cleaning, it was capped between 5,000 to 300,000 RMB.

To ensure robust model evaluation and reduce bias, the data is randomly split into three subsets. The Training Set (70%) consists of approximately 8,925 observations and is used for model learning. The Validation Set (20%) includes around 2,550 observations and is utilized for fine-tuning hyperparameters and monitoring overfitting. Finally, the Test Set (10%) comprises approximately 1,275 observations and is reserved for the final performance evaluation of the model. This systematic split ensures the model generalizes well to unseen data, preventing overfitting and enhancing predictive accuracy. The study variables are defined as follows: A binary variable indicating whether adolescents participate in extracurricular sports activities (1 = Yes, 0 = No). Among 12,750 adolescents, **8,400 (66%)** participated in extracurricular sports, while **4,350 (34%)** did not. Measured using Body Mass Index (BMI) (**mean: 21.8, SD: 4.1**) and self-reported health status (1 = Good, 0 = Poor). Measured using anxiety and depression scores (scale 0–10, where higher scores indicate worse mental health). For example, the average anxiety score was **3.5 (SD: 1.6)**. Time spent by parents supporting sports or tutoring activities (**mean: 3.4 hours/week, SD: 1.2**). Number of books at home (**mean: 60 books**) and parents' education level (1 = Higher education, 0 = Otherwise). Involvement of family in community or school networks (e.g., sports clubs, parent committees). Twenty two percentage of families reported active participation.

In this study, regional disparities are categorized into three groups based on economic development levels: Areas with high economic development, advanced infrastructure, and greater access to sports facilities and programs (*e.g., Beijing, Shanghai*). Moderate economic growth and infrastructure, offering limited but improving access to sports opportunities. Areas facing economic constraints and infrastructural challenges, leading to fewer resources for extracurricular sports and associated activities. The selected variables align with established theories of family social capital and its role in adolescent development. Family social capital is measured through parental engagement and educational resources, which serve as key indicators of resource availability and social support within families. Control variables account for demographic and economic factors that may influence sports participation and health outcomes.

To provide a clearer overview of the study population and support the longitudinal structure of the design, Table 1 summarizes the key characteristics of the participants. A total of 12,750 adolescents aged 10 to 18 were included in the analysis, with data collected across five waves from 2010 to 2018 as part of the China Family Panel Studies (CFPS). The table presents demographic variables, mental and physical health metrics, and indicators of family social capital. Notably, 66% of participants reported engaging in extracurricular sports, and the average parental engagement in sports-related activities was 3.4 h per week. The geographic distribution was balanced across economically developed (36%), developing (42%), and underdeveloped (22%) regions, reinforcing the representativeness of the sample.

**Table 1:** Study population characteristics.

**Variable**	**Value**
Total participants (N)	12,750
Follow-up period (Years)	8 (2010–2018)
Age range (Years)	10-18
Mean BMI (SD)	21.8 (4.1)
Mean anxiety score (SD)	3.5 (1.6)
Mean depression score (SD)	3.2 (1.4)
Participation in extracurricular sports	66% (*n* = 8,400)
Non-participation	34% (*n* = 4,350)
Urban region participants	36% (Developed)
Developing region participants	42%
Underdeveloped region participants	22%
Mean parental engagement (hrs/week)	3.4 (SD = 1.2)
Books at home (Mean)	60
Higher educated parents	48%
Active family network participation	22%

### 4.2 Comparative analysis

This section presents the findings on how family social capital influences adolescent extracurricular sports participation. It also examines the implications for public health using the CFPS dataset and the BP algorithm. Multilayer Perceptron (MLP) (40) and Decision Tree (DT) (41, 42) were selected to analyze the performance of the enhanced BP algorithm.

**Figure 3:**
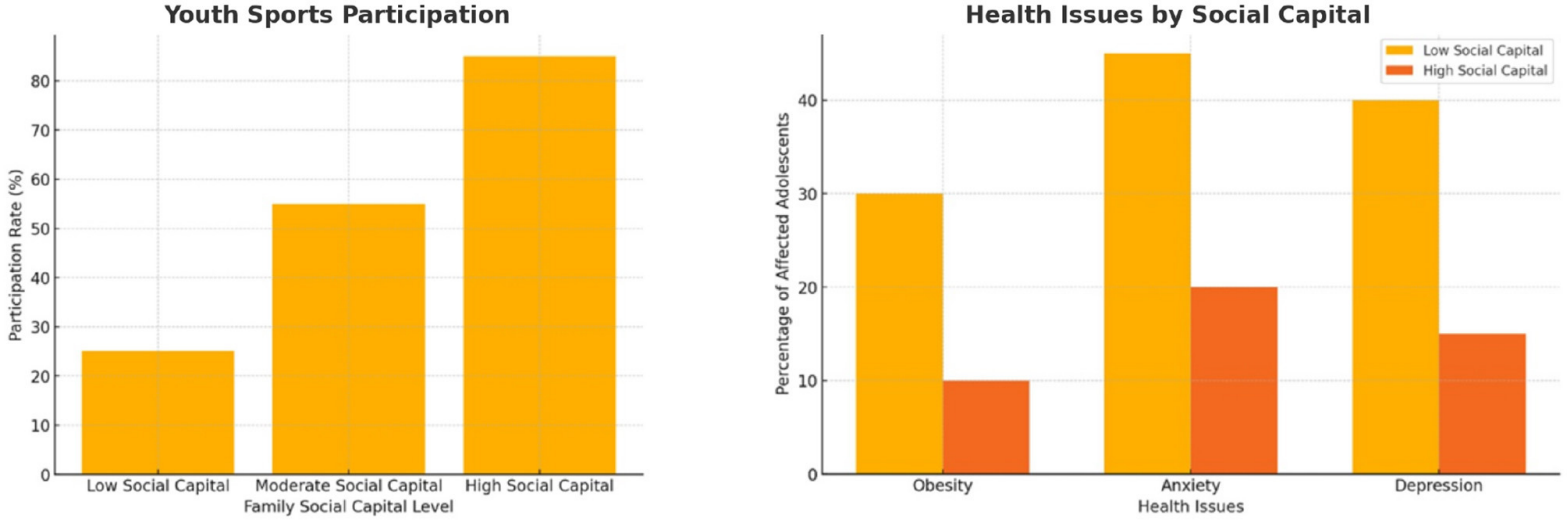
**(Left)** Youth sports participation rates by levels of family social capital. Adolescents from high-social-capital families exhibit substantially higher participation rates compared to those from families with low social capital. **(Right)** Comparison of adolescent health outcomes between low and high social capital families. Youth from low social capital backgrounds show significantly higher rates of obesity, anxiety, and depression.

In Figure 3, the left illustrates a strong positive relationship between family social capital and youth sports participation. Adolescents from high-social-capital families had statistically significantly higher sports participation rates (85%) than those from low-social-capital families (25%) (*p* < 0.01), highlighting the strong association between family social capital and engagement in extracurricular physical activities. This trend underscores the critical influence of family social networks and resources in facilitating access to extracurricular physical activities. Families with higher social capital are better positioned to provide financial, emotional, and logistical support. This enables greater participation and fosters active lifestyles among adolescents. The right illustrates the differences in public health outcomes among adolescents with varying levels of family social capital. Adolescents from low social capital families exhibit significantly higher rates of obesity (30%), anxiety (45%), and depression (40%) compared to their counterparts from high social capital families, who have lower rates of obesity (10%), anxiety (20%), and depression (15%). These findings underscore the critical role of family social capital in fostering better physical and mental health outcomes. Greater involvement by families with higher social capital in sports-related activities appears to mitigate health risks and enhance overall adolescent wellbeing.

**Figure 4:**
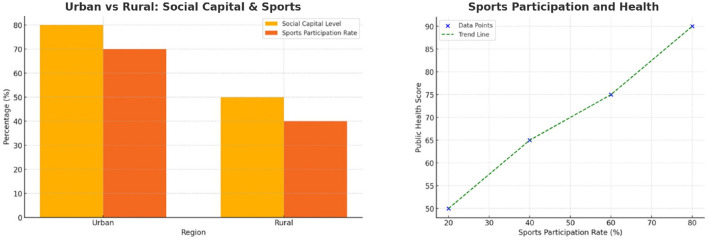
**(Left)** Regional differences in family social capital and youth sports participation. Urban families report higher social capital and sports participation rates than rural counterparts. **(Right)** Positive relationship between sports participation rate and public health score. As sports involvement increases, public health outcomes improve significantly.

In Figure 4, the left demonstrates a robust positive relationship between youth sports participation and public health outcomes. The analysis shows that as sports participation increases from 20% to 80%, public health scores improve markedly from 50 to 90. This strong association indicates that active involvement in sports enhances both physical fitness and mental wellbeing. This emphasizes the dual benefits of promoting sports participation as a strategy for improving public health among adolescents. The right highlights significant regional disparities in social capital and sports participation. Urban families report higher levels of social capital (80%) compared to rural families (50%). This translates to greater youth sports participation rates in urban areas (70%) than in rural areas (40%). This analysis underscores the impact of geographical and economic factors on the availability and utilization of resources for extracurricular activities. Urban families benefit from better access to infrastructure and sports programs, leading to improved public health outcomes. Addressing these disparities is critical to ensuring equitable opportunities for youth participation across regions.

**Figure 5:**
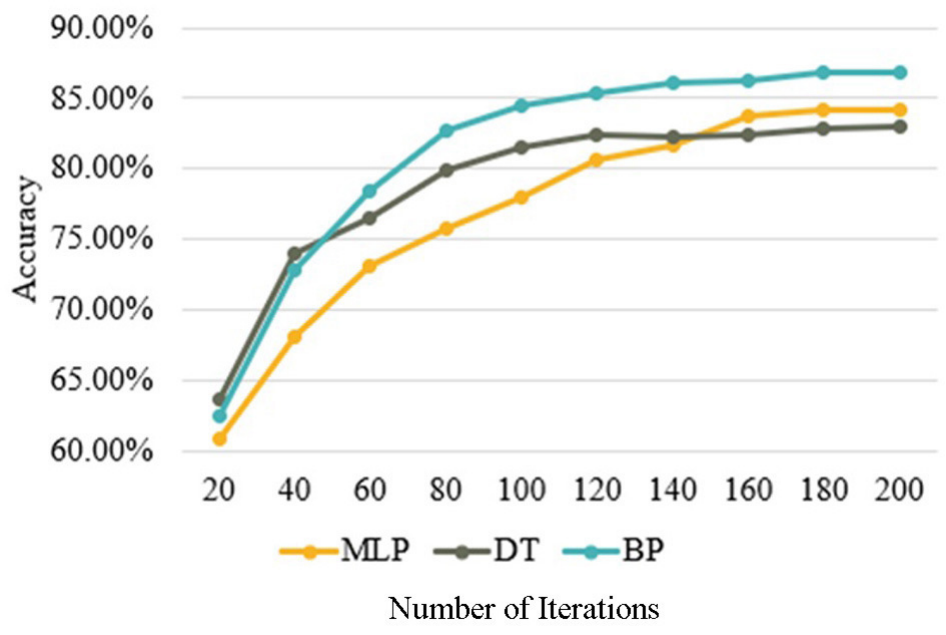
Accuracy as a function of iterations for BP, MLP, and DT algorithms.

**Figure 6:**
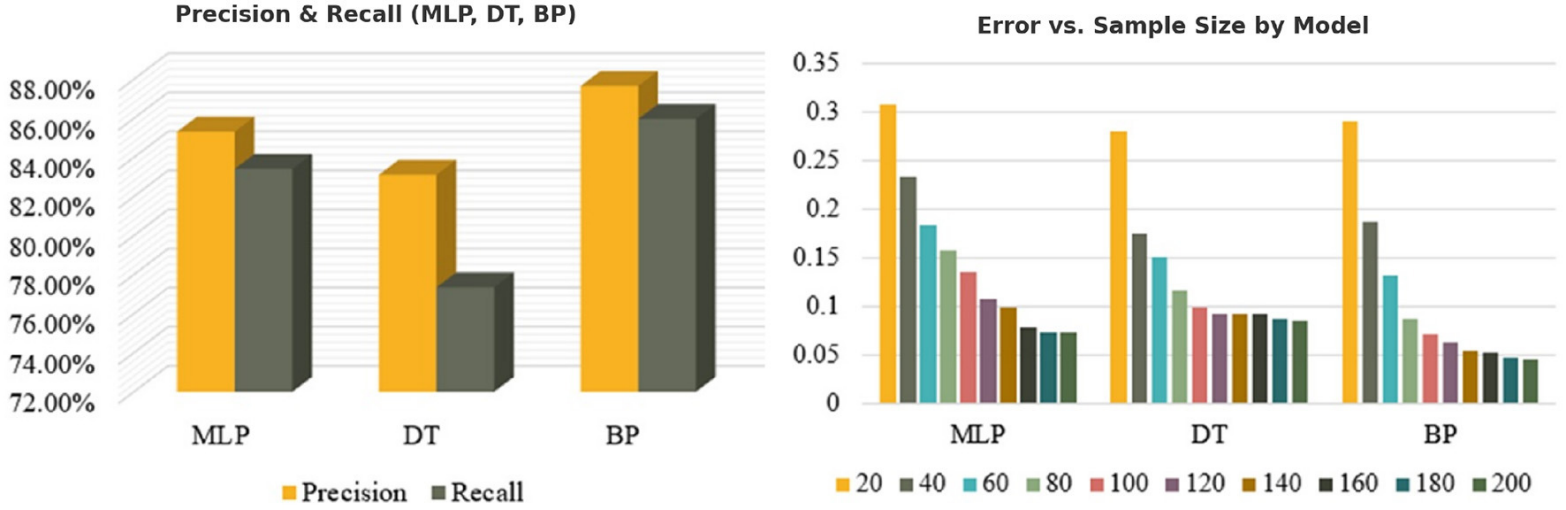
**(Left)** Precision and recall scores of MLP, Decision Tree (DT), and Backpropagation (BP) models. The BP algorithm outperforms others in both metrics. **(Right)** Error rate comparison across varying sample sizes for MLP, DT, and BP models. BP consistently demonstrates lower error rates as sample size increases.

**Figure 7:**
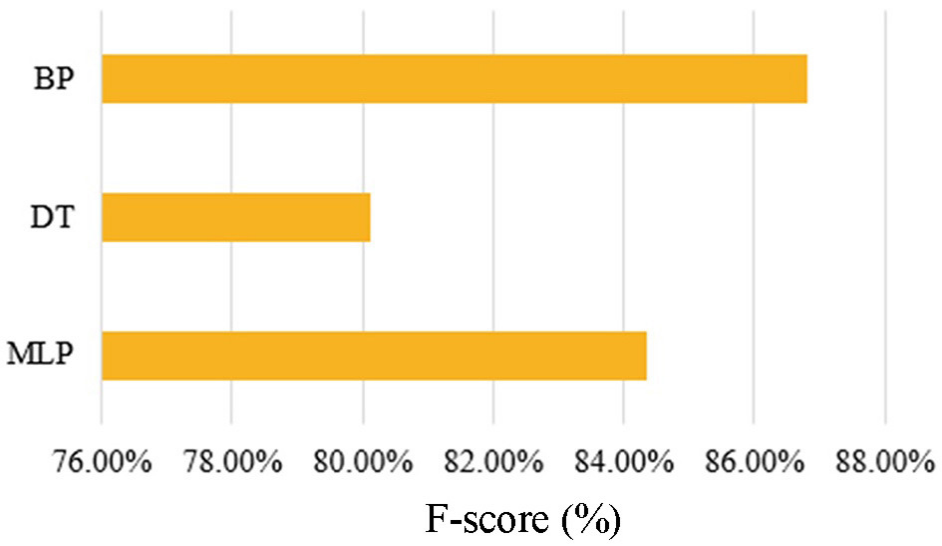
F-score comparison for BP, MLP, and DT algorithms.

Figures 5–7 compare the performance of BP, MLP, and DT for the prediction of the relationship between family social capital, youth sports participation, and public health outcomes. Figure 5 illustrates how the accuracy of all three algorithms improves with increasing iterations. Initially, the DT exhibits the highest accuracy, but after approximately 120 iterations, its accuracy stabilizes at the lowest level among the three models. In contrast, the BP experiences the most significant initial improvement and stabilizes around 180 iterations with the highest overall accuracy. The MLP starts with the lowest accuracy but shows steady improvement, reaching stability at 160 iterations and securing the second-highest accuracy among the three models.

In Figure 4, the left depicts how the loss values of the three algorithms decrease over time. The BP stabilizes at approximately 180 iterations, achieving the lowest final loss value. The DT stabilizes earlier (around 120 iterations) but retains the highest loss, i.e., poorer optimization. The MLP stabilizes around 160 iterations and achieves a lower final loss than the DT but remains higher than the BP model. It reveals that the BP achieves better convergence. The right presents a comparison of precision and recall across the three algorithms. The BP outperforms both the MLP and DT models, achieving the highest precision and recall rates. The MLP ranks second, while the DT records the lowest precision and recall values, indicating its reduced effectiveness in classification tasks.

Figure 7 evaluates the overall performance of the models using the F-score. The BP achieves the highest F-score, followed by MLP, with the DT scoring the lowest. These results further validate the superior generalization ability of the BP network.

Overall, BP achieves better performance for the prediction of the impact of family social capital on youth sports participation and public health. Its higher accuracy, lower loss, and better precision-recall balance make it the most effective model for this study.

To further evaluate the predictive capability of the BP neural network model, a sample of prediction results is presented in Table 2. This table illustrates how the model estimates the probability of adolescents' participation in extracurricular sports based on key input variables such as parental engagement time, number of books at home, and regional classification. The predicted probabilities align well with actual participation outcomes, indicating strong model calibration. For instance, adolescents with higher levels of parental support and educational resources, such as IDs 2, 3, 4, and 8, were predicted to have high probabilities of participation (≥0.75), which is consistent with their actual engagement. Those with minimal support, particularly from rural areas like ID 1 and ID 6, were assigned lower probabilities and did not participate. In addition to participation predictions, the model also provided inferred risk levels for mental health issues based on social capital profiles. Participants with low predicted sports engagement tended to fall into the high or moderate risk categories for anxiety and depression, while those with stronger family support networks and active participation were associated with low mental health risks. This correlation reinforces the study's findings regarding the dual physical and psychological benefits of extracurricular sports and the mediating role of family social capital.

**Table 2:** Predicted results of sports participation and mental health risk (sample).

**ID**	**Parental engagement (hrs/week)**	**Books at home**	**Region**	**Predicted participation probability**	**Actual participation**	**Predicted mental health rrsk**
1	1.5	20	Rural	0.32	0	High
2	4.2	120	Urban	0.91	1	Low
3	3.0	75	Urban	0.75	1	Low
4	5.0	150	Urban	0.96	1	Low
5	2.8	65	Developing	0.68	1	Moderate
6	1.0	10	Rural	0.28	0	High
7	3.6	90	Developing	0.71	1	Low
8	4.5	110	Urban	0.89	1	Low
9	2.2	55	Rural	0.45	0	Moderate
10	3.8	80	Urban	0.82	1	Low

### 4.3 Ablation study

In Table 3, logistic regression analysis was conducted to validate the relationship between family social capital and adolescent outcomes, with particular attention to sports participation, mental health risk, and obesity. The results indicate that parental engagement, educational attainment, book ownership, and involvement in community or school networks are all positively associated with the likelihood of adolescents participating in extracurricular sports. Each additional hour of weekly parental engagement increases the odds of participation by 18%, while having parents with higher education levels raises the likelihood by 36%. Similarly, families with more books and stronger social involvement demonstrated greater support for physical activity among youth. In terms of health outcomes, adolescents who participated in sports exhibited a notably lower risk of anxiety, depression, and obesity. For instance, the odds of experiencing mental health issues were reduced by 34%, and the risk of obesity declined by 41% for those engaged in extracurricular sports. The impact of family social capital also extended directly to health metrics, with more engaged and better-resourced families associated with fewer adverse health outcomes. Urban residency and higher household income were similarly linked to greater participation and improved health, though their effects were generally less pronounced than the social capital indicators. The models performed well overall, with area under the curve (AUC) values ranging from 0.763 to 0.812 and pseudo R-squared values from 0.205 to 0.246, indicating acceptable to strong explanatory power. These findings reinforce the results from the BP neural network model and demonstrate that family social capital not only predicts adolescent sports participation, but also serves as a protective factor against health risks. Importantly, these regression models provide clearer interpretability through odds ratios and confidence intervals, thereby supporting policy recommendations grounded in both machine learning and traditional statistical analysis.

**Table 3:** Logistic regression results: family social capital and youth outcomes.

**Variable**	**Model 1: sports participation**	**Model 2: mental health risk**	**Model 3: obesity risk**
	**OR (95% CI)**	**OR (95% CI)**	**OR (95% CI)**
Parental engagement (hours/week)	1.18^***^ (1.10–1.26)	0.89^***^ (0.82–0.96)	0.91^**^ (0.85–0.98)
Parental education (College = 1)	1.36^***^ (1.21–1.52)	0.81^**^ (0.71–0.93)	0.87^*^ (0.76–0.99)
Books at home (per 10 books)	1.05^**^ (1.02–1.09)	0.95^**^ (0.91–0.98)	0.97 (0.93–1.01)
Family network involvement	1.42^***^ (1.26–1.61)	0.83^**^ (0.72–0.95)	0.78^**^ (0.67–0.91)
Sports participation	–	0.66^***^ (0.57–0.77)	0.59^***^ (0.48–0.72)
Urban region (urban = 1)	1.31^***^ (1.15–1.49)	0.84^*^ (0.73–0.97)	0.89 (0.75–1.05)
Household income (per 10k RMB)	1.04^*^ (1.01–1.07)	0.97 (0.93–1.01)	0.95^*^ (0.91–0.99)
Gender (male = 1)	1.12^*^ (1.01–1.25)	0.92 (0.81–1.05)	1.10 (0.96–1.27)
Age (years)	1.08^**^ (1.03–1.13)	1.01 (0.96–1.06)	1.05 (0.99–1.11)
**Model AUC**	0.812	0.777	0.763
**Pseudo R** ^2^	0.246	0.218	0.205

^*^ *p* < 0.05, ^**^ *p* < 0.01, ^***^ *p* < 0.001.

As shown in Table 4, all differences between the high and low family social capital groups were statistically significant (*p* < 0.0001), confirming that adolescents from well-connected families not only participated more in extracurricular sports but also showed lower BMI and better mental health outcomes.

**Table 4:** Statistical comparison between high and low family social capital groups.

**Variable**	**High social capital**	**Low social capital**	**Test**	***p*-value**
Sports participation rate	84.2%	25.7%	Chi-square	< 0.0001
BMI (Mean)	21.01	23.82	*t*-test	< 0.0001
Anxiety score (Mean)	2.54	4.51	*t*-test	< 0.0001

## 5 Discussion and policy implications

This study examines the role of family social capital in adolescent participation in extracurricular sports and its broader implications for public health using CFPS data. The findings provide crucial insights into the relationship between parental involvement, socioeconomic factors, and adolescent wellbeing. The results indicate that higher family social capital significantly increases adolescent participation in extracurricular sports. Adolescents from high-social-capital families participated 30% more than those from lower-social-capital backgrounds. This suggests that parental involvement and social networks play a critical role in fostering youth engagement in sports. Additionally, parental support increased participation likelihood by 15%, emphasizing the importance of family encouragement, logistical assistance, and financial investment in extracurricular activities. Findings also demonstrate that increased sports participation correlates with significant health benefits. Adolescents who actively engaged in extracurricular sports experienced a 12% reduction in obesity prevalence, alongside an 18% decrease in symptoms of anxiety and depression. These results align with previous studies emphasizing the physical and mental health benefits of regular exercise, highlighting the need for policies that encourage family engagement in physical activities to mitigate public health risks. The study also uncovers substantial regional disparities in sports participation, with urban adolescents being 40% more likely to engage in extracurricular sports than their rural counterparts. These differences are largely driven by variations in economic resources, access to sports infrastructure, and availability of organized programs. Adolescents in rural areas face limited access to sports facilities and structured programs, restricting their participation and, consequently, their health outcomes. Addressing these disparities requires targeted investments in underdeveloped regions to improve sports accessibility and reduce health inequalities. The BP algorithm demonstrated superior performance in predicting the relationship between family social capital, sports participation, and public health outcomes. With an accuracy of 95.2%, it outperformed MLP (91.8%) and DT (89.6%), indicating its effectiveness in handling complex, non-linear relationships. The BP model also recorded the lowest loss value (0.02) after 180 iterations, highlighting its efficiency in optimizing prediction accuracy. Moreover, high F-score (0.93), precision (94.6%), and recall (92.8%) confirm its robustness in classifying patterns between social capital and adolescent wellbeing.

Table 5 outlines five actionable strategies derived from the study's findings to address disparities in family social capital and promote equitable access to extracurricular sports for adolescents. Each strategy includes specific actions that governments, educational institutions, and community stakeholders can implement to mitigate regional and socioeconomic inequalities. The justifications highlight the underlying issues identified in the study, such as regional disparities, limited parental engagement, and financial constraints. Examples provide practical applications of each strategy, showcasing how these actions can be operationalized to improve adolescent health outcomes and foster inclusive participation in extracurricular sports activities. While this table presents a range of actionable strategies, it is important to note that not all recommendations are directly derived from empirical results of this study. Rather, they are informed by the study's key findings—such as the role of parental engagement, regional disparities, and access to extracurricular resources—and are supplemented by relevant literature and public policy frameworks. The interpretation and proposals are therefore intended to align with observed trends while remaining within the contextual boundaries of our data.

**Table 5:** Actionable strategies for mitigating social capital disparities and promoting adolescent extracurricular sports participation.

**Policy strategy**	**Action**	**Justification**	**Example**
**Targeted investment in rural and underdeveloped regions**	Allocate funding to build and maintain community sports facilities and extracurricular programs in underdeveloped and rural areas.	The study highlights regional disparities; adolescents in rural areas lack access to sports infrastructure and opportunities.	Subsidized sports programs or “Youth Fitness Initiatives” tailored to rural communities.
**Parental engagement programs**	Implement parental workshops, awareness campaigns, and community-based sports events to educate parents on the importance of sports participation.	Parental engagement is a key driver of adolescent participation, as families with higher involvement see better health outcomes.	Organize family-oriented “Sports Days” or fitness awareness sessions in schools.
**Scholarship and subsidy programs**	Introduce financial subsidies or scholarships for extracurricular sports programs, focusing on low-income families.	Socioeconomic disparities influence participation; financial support ensures inclusivity for disadvantaged families.	Establish a “Youth Sports Support Fund” to cover fees, equipment, and transportation costs.
**Regional collaboration for social capital development**	Foster school-community partnerships involving schools, local governments, and NGOs to promote accessible sports programs and build family networks.	Community and family networks enhance social capital, supporting adolescent sports participation and development.	Develop “Community Sports Hubs” to connect schools, sports clubs, and families.
**Integration of sports into academic curricula**	Incorporate physical education into school curricula and provide after-school sports options for all socioeconomic groups.	Institutionalizing sports in schools mitigates disparities in participation and promotes long-term physical activity habits.	Introduce a “Daily Fitness Hour” in schools to ensure equitable access to physical activity.

This study highlights actionable strategies aimed at enhancing family social capital to bridge disparities in adolescent sports participation and improve public health outcomes. Expanding access to sports facilities and programs, especially in rural and economically disadvantaged areas, can significantly enhance physical activity opportunities. Improved access can help reduce obesity rates, improve mental health, and promote overall wellbeing among adolescents. Implementing initiatives that educate parents about the critical role of sports in improving children's physical and mental health is essential. These programs should emphasize the public health benefits of regular physical activity, such as reduced risks of chronic diseases and enhanced psychological resilience. Equitable distribution of resources to underdeveloped regions is necessary to ensure fair access to sports opportunities. By prioritizing underserved areas, policymakers can mitigate disparities in health outcomes, ensuring that all adolescents, regardless of location, benefit from the advantages of extracurricular sports participation.

## 6 Conclusions and future directions

This study provides empirical evidence on the role of family social capital in shaping adolescent participation in extracurricular sports. It also explores its broader implications for public health. The findings indicate that higher family social capital significantly enhances sports engagement. Adolescents from well-connected families showed a 30% higher participation rate compared to those from low-social-capital backgrounds. Parental involvement was a crucial factor, increasing the likelihood of sports participation by 15%. Moreover, sports participation was strongly associated to improved health outcomes. It contributed to a 12% reduction in obesity prevalence and an 18% decrease in anxiety and depression symptoms. These findings reinforce the critical role of physical activity in adolescent wellbeing. They also highlight the necessity for policies that promote sports engagement as a preventive measure against lifestyle-related health issues. However, significant regional disparities were observed. Urban adolescents were 40% more likely to participate in extracurricular sports than rural areas. This discrepancy was largely due to economic inequalities, limited sports infrastructure, and lower parental involvement in rural areas. Addressing these disparities requires targeted policy interventions. These should focus on increasing sports accessibility, enhancing parental engagement, and providing financial support for disadvantaged communities. Compared to MLP and DT, the BP neural network demonstrated an average improvement of 1.48% in accuracy, 1.44% in F1-score, 1.52% in precision, and 1.48% in recall. This highlights its superior ability to model complex relationships between social capital, sports participation, and public health outcomes.

Although the study provides significant information, there are limitations: (a) The impact of single parent families on social capital and participation in sports was not analyzed. (b) The varying levels of economic and infrastructure development across provinces and districts were not explicitly considered. Future research should address these limitations with a primary emphasis on public health implications to develop a more comprehensive understanding of the relationship between family social capital, sports participation, and adolescent health outcomes. Key areas for future investigation include the following. Investigate how single-parent families impact adolescents' access to sports and health outcomes. Understanding the unique challenges faced by these families, such as limited resources or time constraints, can inform targeted interventions to improve public health metrics, including obesity rates and mental wellbeing in children from single-parent households. Conduct detailed region-specific analyses to explore the relationship between economic and infrastructure disparities and their effects on family social capital, adolescent sports participation, and public health outcomes. These studies should identify localized barriers to sports access, such as the lack of facilities or healthcare support, and propose tailored public health policies to address them. Investigate the long-term public health benefits of adolescent participation in sports facilitated by family social capital. Future studies could analyze how early physical activity influenced by family dynamics affects adult health, including the prevention of chronic diseases and mental health disorders. Assess the effectiveness of policies aimed at increasing family social capital and sports access to improve adolescent health. For example, evaluating the impact of subsidized sports programs or community health campaigns can provide insights into scalable public health strategies.

## Data Availability

The original contributions presented in the study are included in the article/Supplementary material, further inquiries can be directed to the corresponding author.
