# Correction: A study on the influence of family social capital on participation in adolescent extracurricular sports and public health

**DOI:** 10.3389/fpubh.2025.1659570

**Published:** 2025-08-12

**Authors:** Wenjie Sui, Miaohong Huang

**Affiliations:** ^1^College of Physical Education, Huaqiao University, Quanzhou, China; ^2^Xiamen Institute of Technology, Xiamen, China

**Keywords:** public health, obesity, anxiety, depression, educational resources, CFPS data, family social capital, youth extracurricular sports

Author Wenjie Sui was erroneously assigned as corresponding author. The correct corresponding author is Miaohong Huang.

The original version of this article has been updated.

