# Supplementary material for: A study on the influence of family social capital on participation in adolescent extracurricular sports and public health
Source: Front Public Health. 2025 Jun 25;13:1515522. doi: 10.3389/fpubh.2025.1515522 (PMC12238025; doi:10.3389/fpubh.2025.1515522)
Supplement: Supplementary file 1 [file Data_Sheet_1.pdf]

Supplementary Material

Table S1 provides a comparison of the strengths and limitations of the BP, DT, and MLP models. It highlights why the BP model was chosen for this study, given its ability to handle complex, non-linear relationships and large datasets. While DT offers interpretability and efficiency for smaller data, it struggles with complexity. MLP is a capable alternative but less effective for larger datasets without optimization. Therefore, this table enhances the robustness of the discussion and clarifies the rationale behind the model choice.

Table S1. Strengths and Limitations of BP Neural Network, Decision Tree, and MLP

Model	Strengths	Limitations
**BP**	• Handles complex, non-linear relationships effectively.<br>• Learns intricate patterns and hierarchical feature interactions automatically.<br>• Scalable and robust with large datasets.	• Computationally intensive and time-consuming for large datasets.<br>• Requires careful hyperparameter tuning to avoid overfitting.<br>• Less interpretable compared to simpler models.
**DT**	• Easy to interpret and visualize, providing clear decision rules.<br>• Computationally efficient, with quick training times.<br>• Works well with smaller datasets.	• Struggles with non-linear relationships and complex interactions.<br>• Prone to overfitting without pruning or ensemble methods.<br>• Performance plateaus when handling large or complex data.
**MLP**	• Similar to BP, capable of handling non-linear relationships and feature interactions.<br>• Easier to implement for smaller datasets.<br>• Flexible and adaptable for moderate-sized problems.	• Can suffer from the vanishing gradient problem in deep networks without optimization.<br>• Requires extensive tuning of parameters to achieve high performance.<br>• Less efficient for large datasets compared to BP.
